# Cognitive tests to help diagnose dementia in symptomatic people in primary care and the community

**DOI:** 10.3399/bjgp18X695249

**Published:** 2018-03

**Authors:** Sam Creavin, Susanna Wisniewski, Anna Noel-Storr, Sarah Cullum, on behalf of the MMSE review team

**Affiliations:** Population Health Sciences, Bristol Medical School, University of Bristol, Bristol, UK.; Cochrane Dementia and Cognitive Improvement Group, Oxford University, Oxford, UK.; Cochrane Dementia and Cognitive Improvement Group, Oxford University, Oxford, UK.; Department of Psychological Medicine, School of Medicine, Faculty of Medical and Health Sciences, University of Auckland, Auckland, New Zealand.

## INTRODUCTION AND BACKGROUND

What brief cognitive test should a busy GP use when trying to assess someone who might have dementia? The menu of choices is long; one review found 11 options.^1^

The Cochrane Dementia and Cognitive Improvement Group (CDCIG) is conducting a series of reviews to evaluate the evidence of a range of tests for diagnosing dementia. To date, reviews have been published addressing the accuracy of two tests in primary care: the Informant Questionnaire for Cognitive Disorders in the Elderly (IQCODE) and the Mini Mental State Examination [MMSE]. Reviewers found only one study that investigated the use of the IQCODE in primary care,^2^ and six that investigated the use of the MMSE.^3^

A review of the Montreal Cognitive Assessment [MoCA] found no studies that evaluated the accuracy of the test in primary care.^4^ Reviews are underway for the Mini-Cog and AD-8 tests (see http://dta.cochrane.org/reviews-and-protocols-crg).

## REVIEW OF EVIDENCE FOR THE DIAGNOSTIC ACCURACY OF THE IQCODE IN PRIMARY CARE

The IQCODE is a structured informant questionnaire; 26-item and 16-item versions exist and scores range from 1 (no impairment) to 5 (more impairment).^4^

In the one study that investigated the use of the IQCODE at a threshold of 3.2 in primary care the sensitivity was 100% and specificity 76%, whereas at a threshold of 3.7 the sensitivity was 75% and specificity 98%.^2^

## REVIEW OF EVIDENCE FOR THE DIAGNOSTIC ACCURACY OF THE MMSE IN PRIMARY CARE

The MMSE is one of the oldest and therefore most well-known cognitive tests for dementia, though it may now be less likely to be used by clinicians due to copyright fees. The Cochrane review identified 24 310 articles, of which 317 full-text articles were reviewed and 70 records (referring to 48 studies) were included. Of the 48 included studies, six were in primary care and 42 were in unselected members of the general community aged >65 years.

## PRIMARY CARE STUDIES

Of the six primary care studies, two were conducted in patients who were presenting with symptoms of dementia and four were in patients attending primary care regardless of concerns about dementia. The prevalence of dementia would be expected to be higher in people with symptoms and this would affect the accuracy of the test.

No study was judged as being at high risk of bias in more than two of four areas.

**Figure 1.**
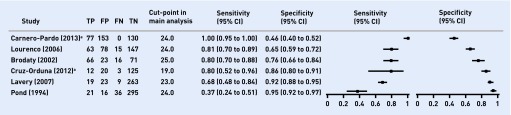
***Forest plot of primary care studies.****3* ^a^**Carnero-Pardo 2013 and Cruz-Orduna 2012 were in symptomatic patients. CI = confidence interval. FN = false negative. FP = false positive. TN = true negative. TP = true positive.**

Figure 1 presents the Forest plot for the sensitivity and specificity of studies in primary care. There were too few studies to perform meta-analysis of the accuracy of any one cut-point.

### Symptomatic patients

In symptomatic patients (that is, patients presenting with memory problems) in primary care the accuracy of the MMSE for the diagnosis of dementia was available for 9 cut-points (17 to 25 inclusive) in two different studies. Only one study (Carnero-Pardo 2013) reported the traditional cut point of 24, with an accuracy of sensitivity 1.00 (95% CI = 0.95 to 1.00) and specificity 0.46 (95% CI = 0.40 to 0.52).

### Asymptomatic patients

In asymptomatic patients the accuracy of the MMSE for the diagnosis of dementia was available for 9 cut-points (18 to 26 inclusive) in four different studies. At the traditional cut- point of 24, Lourenco 2006 (Brazil) reported a sensitivity of 0.81 (95% CI = 0.70 to 0.89) and specificity of 0.65 (95% CI = 0.59 to 0.72), whereas Pond 1994 (Australia) reported a sensitivity of 0.37 (95% CI = 0.24 to 0.51) and specificity of 0.95 (95% CI = 0.92 to 0.97). The prevalence of dementia in Lourenco 2006 was higher than in other studies and this may explain the difference in findings.

## WHAT ABOUT SCREENING?

Screening for dementia in the general population is not currently recommended by the UK screening committee.^5^

## WHAT ISSUES WITH COGNITIVE TESTS SHOULD GPs BE AWARE OF?

Specific patients may present problems; in particular, the presence of sensory impairment, coexisting morbidity, and learning and/or language difficulties may make the assessment particularly challenging.

GPs should be aware that it is possible that someone might have dementia even with a very high ‘score’ that is well above the normal cut-point to identify cognitive impairment. Education has an important confounding effect on the scores obtained on brief cognitive tests and diagnostic criteria for dementia are clear that the relevant finding is a decline in cognition from a previous higher level that interferes with daily life, and is not better explained by another mental disorder.

The accuracy of brief cognitive tests for identifying less common cognitive disorders, such as frontotemporal dementia and Lewy body dementia, is not established in primary care.

## WILL FUTURE RESEARCH HELP?

A prospective test accuracy study based in primary care is currently under way that will evaluate the accuracy of a range of tests for diagnosing dementia in a symptomatic group of people recruited from primary care.^6^

## SO WHAT SHOULD THE BUSY GP DO?

A guide for clinicians from the Alzheimer’s Society^7^ recommends the use of the Abbreviated Mental Test Score (<5 minutes), GPCOG (5 minutes), or Mini-Cog (2–4 minutes) as part of the assessment for identifying, dementia in primary care. The authors preference in practice is to use the GPCOG because it assesses recall and visuospatial skills, together with an informant interview, and is brief. The GPCOG also asks particularly about difficulties with managing medications, finances, and gadgets as there is evidence that people with dementia may struggle with these tasks.^8^^,^^9^ Younger patients (<70 years) with symptoms that are acknowledged by an informant present a particular challenge, and in practice the authors would exclude affective disorder and then have a low threshold for seeking advice. For older people (>90 years) with multimorbidity (for example, in a nursing home) it might be more appropriate to focus on managing distress and advance care planning. No cognitive test appears to have sufficient accuracy, by itself, to rule in or rule out dementia. Given the limitations of existing tests and the available evidence, GPs should also value their own judgement based on clinical history and discussion with an informant.

## AN IMPORTANT REMINDER

It is important that clinicians assess the risks to the patient and others, for example, with medications (including anticoagulants), cooking, fires, machinery, or driving.
